# Development of FSW Process Parameters for Lap Joints Made of Thin 7075 Aluminum Alloy Sheets

**DOI:** 10.3390/ma17030672

**Published:** 2024-01-30

**Authors:** Piotr Lacki, Anna Derlatka, Wojciech Więckowski, Janina Adamus

**Affiliations:** 1 Faculty of Civil Engineering, Czestochowa University of Technology, J.H. Dabrowskiego 69 Str., 42-201 Czestochowa, Poland; anna.derlatka@pcz.pl (A.D.); janina.adamus@pcz.pl (J.A.); 2 Faculty of Mechanical Engineering and Computer Science, Czestochowa University of Technology, J.H. Dabrowskiego 69 Str., 42-201 Czestochowa, Poland; wojciech.wieckowski@pcz.pl

**Keywords:** artificial neural networks, friction stir welding, aluminum alloys, lap joints, thin sheets

## Abstract

The article describes machine learning using artificial neural networks (ANNs) to develop the parameters of the friction stir welding (FSW) process for three types of aluminum joints (EN AW 7075). The ANNs were built using a total of 608 experimental data. Two types of networks were built. The first one was used to classify good/bad joints with MLP 7-19-2 topology (one input layer with 7 neurons, one hidden layer with 19 neurons, and one output layer with 2 neurons), and the second one was used to regress the tensile load-bearing capacity with MLP 7-19-1 topology (one input layer with 7 neurons, one hidden layer with 19 neurons, and one output layer with 1 neuron). FSW parameters, such as rotational speed, welding speed, and joint and tool geometry, were used as input data for ANN training. The quality of the FSW joint was assessed in terms of microstructure and mechanical properties based on a case study. The usefulness of both trained neural networks has been demonstrated. The quality of the validation set for the regression network was approximately 93.6%, while the errors for the confusion matrix of the test set never exceeded 6%. Only 184 epochs were needed to train the regression network. The quality of the validation set was approximately 87.1%. Predictive maps were developed and presented in the work, allowing for the selection of optimal parameters of the FSW process for three types of joints.

## 1. Introduction

**Figure 1:**
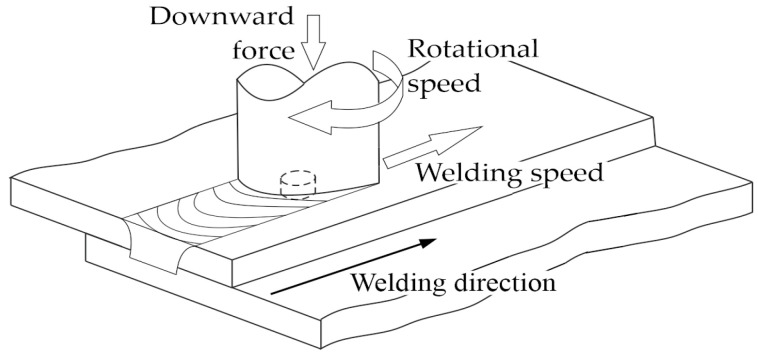
Diagram of the FSW process.

Friction stir welding (FSW) is a solid-state welding process that is used to join materials, typically aluminum and other non-ferrous metals, without melting them. It was developed in the early 1990s and owes its popularity to the ability to create high-quality, defect-free welds with challenging materials. The idea of the FSW is given in Figure 1.

The first patent from 1991 was primarily concerned with controlling the position and speed of the tool during the FSW process. Over the years, FSW technology has evolved considerably. Researchers and engineers have conducted extensive studies and experiments to refine the process and realize its full potential. The beginnings of FSW primarily involved basic control over tool movement. As research progressed, efforts were made to optimize and improve process parameters. This included a more advanced understanding of tool rotation speed, plunge depth, traverse speed, and axial force to achieve greater control over the flow of the plasticized material, resulting in better weld quality and mechanical properties.

### 1.1. Methods of Controlling the FSW Process

In recent years, many different methods have been proposed to control the FSW process to ensure good-quality welds. One of them is tool temperature control, used for regulating the rotation speed of the tool. The authors of work [1] indicate that temperature control in FSW allows obtaining better weld properties. In the works [2,3], it is emphasized that careful selection of FSW parameters allows the elimination of defects, such as voids or zigzag lines, maximizing mechanical properties and regulating the location of fracture in friction stir-welded joints. In the publication [4], to obtain optimal parameters, a response surface (second order) regression model was developed to achieve ultimate tensile strength (UTS) of the joints as a function of tool spindle speed, tool transverse speed, downward force, and tool tilt angle. To ensure the reliability and accuracy of the developed model, a series of statistical tests were conducted. These tests included analysis of variance (ANOVA), the F-ratio, and the assessment of actual and adjusted R-squared (R2) values. These statistical analyses were used to evaluate the suitability and effectiveness of the regression model in representing the true relationship between the input parameters and the UTS of the joints. In the work [5], one-way ANOVA analysis was also performed to analyze the factors influencing weld defects. The application of Taguchi methodology in combination with ANOVA enables the assessment of the statistical importance of various process parameters in relation to the resulting outcomes. In the research described in reference [6], a comprehensive statistical optimization was carried out using a series of experiments. This optimization process focused on evaluating the ultimate tensile strength and elongation of dissimilar joints (AA5454 and AA7075) produced by friction stir welding.

### 1.2. Parameter Optimization Using a Machine Learning Approach

Artificial intelligence (AI) offers possibilities for predicting and optimizing FSW process parameters. Particularly, machine learning techniques can be employed to develop predictive models [7,8,9,10,11]. These models can analyze historical data related to FSW, considering factors, such as tool speed and force and material properties, to predict optimal process parameters for the desired outcomes, like weld strength, quality, and defect minimization. Study [8] discusses the development of an integrated prediction model for ultimate tensile strength (UTS), maximum hardness (MH), and heat input (HI) in friction stir welding of AA-7075. The aim of the work was to improve FSW welding procedures by incorporating four control parameters: tilt angle, rotation speed, welding speed, and shoulder diameter. An increasing number of publications present the combination of design of experiments (DOE) and machine learning (ML) as a methodology to collect and analyze data on a specific industrial phenomenon. In this context, article [12] discusses the choice of design in relation to the ML model performances. It is important to note that the effectiveness of AI in predicting and optimizing FSW parameters depends on the quality and quantity of available data, the choice of AI algorithms, and the specific goals of the optimization. In paper [13], it was emphasized that predicting the mechanical properties of FSW joints involves a high level of non-linearity and ambiguity. Therefore, artificial neural networks could be employed to predict the load bearing and efficiency of FSW joints. The works [14,15] discuss the use of machine learning methods to analyze the results of experimental test in the regression issue. Based on the analysis of backpropagation neural networks, support vector regression, Gaussian process regression, and random forest (RF), the developed backpropagation neural network model showed greater accuracy and excellent capabilities compared to other methods.

Whether leveraging machine learning techniques, deep learning architectures, or hybrid approaches, the algorithms profoundly influence the AI system’s ability to discern patterns, generalize insights, and adapt to the complexity of the FSW process. Furthermore, continuous updates to the AI model based on new data and ongoing feedback from the FSW process can enhance its predictive capabilities over time. The successful integration of AI into FSW optimization strategies necessitates a collaborative effort between domain experts, data scientists, and engineers to ensure that the AI system aligns with the evolving dynamics of the welding process.

Therefore, in the present research, a machine learning-based approach was proposed to predict classification and regression of friction stir welding process parameters. A backpropagation neural network was developed to describe the complicated relationship between the welding parameters and quality of the joints. The classification was based on the good/bad connector criterion. The good/bad joint criterion was determined taking into account the required load-bearing capacity of the joint and microstructure assessment. The regression artificial neural network was designed for prediction of the load-bearing capacity of joints. To demonstrate the effectiveness of the proposed approach, the prediction results were demonstrated using the fitted surfaces. The visualizations gave a better understanding of the influence of FSW parameters on the quality of joints.

## 2. Materials and Methods

The ability of FSW to produce high-strength and high-quality welds without the need for melting the base materials has made it a valuable technology for joining high-strength aluminum alloys like EN AW 7075. Due to its wide range of applications, this aluminum alloy is still the subject of many studies [16,17,18]. EN AW 7075 is a high-strength aluminum alloy [19] known for its excellent mechanical properties, which makes it attractive for various aerospace and automotive applications [18,20,21,22]. FSW offers a promising way to join EN AW 7075 and other similar high-strength aluminum alloys.

**Figure 2:**
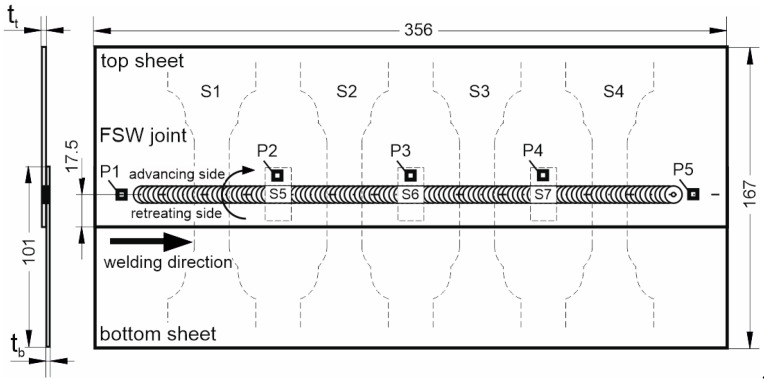
Geometry of the FSW joint, mm.

During the experiment, FSW lap joints with a length of 325 mm were produced from the sheet metal with dimensions of 356 mm × 101 mm. Sheet metals with thicknesses of 1.6 mm, 1.0 mm, and 0.8 mm were used for the tests. A diagram of the control joint along with the configuration of the thickness of the joined sheets is presented in Figure 2.

The welding process was carried out on the upper side of the sheet, the thickness of which is given in Table 1.

The sheet made of EN AW 7075 alloy [23] clad on both sides was used in the study. The experimentally determined chemical composition of analyzed sheet is presented in Table 2.

**Figure 3:**
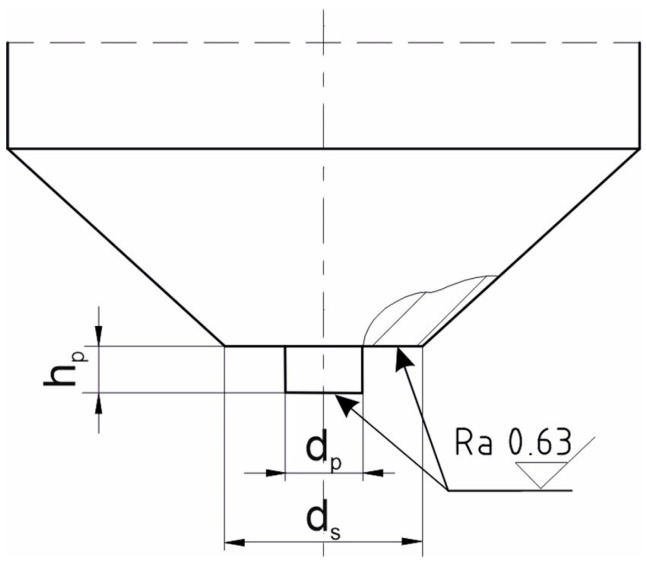
Geometry of the working part of the FSW tool used for testing.

Tools designed by the authors of the work, with a flat shoulder and a cylindrical-shaped pin with a smooth surface, were used to produce the joints. Different shoulder diameters and different pin sizes were tested. The geometry of the working part and the set of tools used for testing are shown in Figure 3.

The dimensions of the working parts of the FSW tools are shown in Table 3. T1–T6 tools were used for producing J1 joints. T2 and T7–T10 tools were used for J2 joints, while J3 joints were made using T11–T14 tools.

Experimental studies were conducted with different parameters of the welding process, i.e., with a variable rotational tool speed n of 700–6000 rpm and a variable welding speed v of 50–2100 mm/min.

The joints were produced using a CNC machining center with the following parameters: number of controlled axes, 4; X, Y and Z-axis travel, 1040/600/500 mm; table surface area, 1250 × 600 mm; max. table load, 600 kg; spindle speed, 12,000 rpm; feed rate, 0–40,000 mm/min; and spindle motor power, 19 kW.

**Figure 4:**
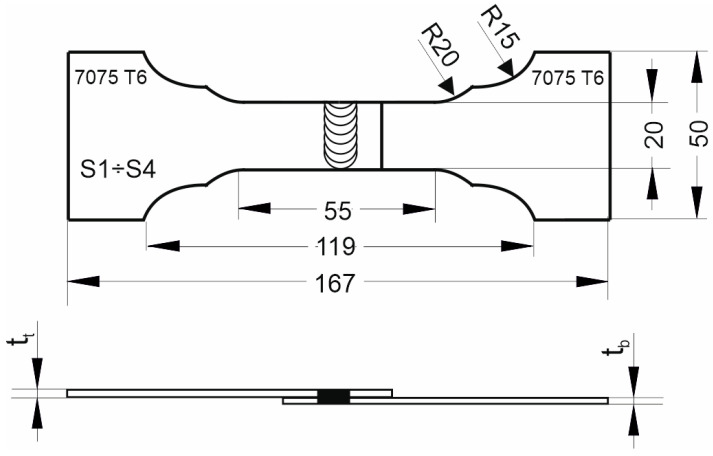
Dimensions of specimens for the strength test, mm.

Four specimens S1–S4 for strength tests and three specimens S5–S7 for metallographic tests were cut out from the prepared joints, as marked in Figure 2. Samples were cut out using abrasive water jet cutting. The geometry of the specimens for strength tests is shown in Figure 4. The specimens were subjected to a controlled tension until failure using a Zwick Z050 testing machine with a tensile speed of 2 mm/min. The quality of FSW joints was assessed by evaluating the load-bearing capacity and the microstructure on the joint cross-section. The load-bearing capacity was determined as the average value of the force transferred by S1–S4 samples.

Joints with a load-bearing capacity of not less than 70% of the load-bearing capacity of the base material for the weaker (thinner) sheet metal and joints free of defects, such as hooking, cold lap, kissing bond, heterogeneous stirring and thinning, were considered to be of good quality.

## 3. Development of Process Parameters Using Machine Learning

Machine learning using neural networks was implemented to develop the parameters of the FSW process. Data from the experiments were used to build the networks. Two types of networks were built. The first one was used to classify good/bad joints, and the second one was used to regress the tension load capacity.

A total of 608 samples were used to build neural network classification and regression, including 98 samples of joints made of 1.6 and 0.8 mm thick sheets, 228 samples of joints made of two 1.0 mm thick sheets, and 282 samples of joints made of 1.0 and 0.8 mm thick sheets. In each joint type, the samples were divided into three groups: 70% of the samples were used for teaching the network (learning set), 15% of the samples were used to test the learned networks in order to select the network (testing set), and 15% of the samples were used for validation of the joint quality (validation set). The summary is presented in Table 4.

When starting to design the networks, the most common rule of thumb presented in [24,25], which states that the sample size needs to be at least a factor 10 times the number of weights in the network, was used. However, the exact selection of the network was made based on the analysis of networks differing in hyperparameters.

### 3.1. Classification

The random oversampling method was performed using the learning and testing sets for each type of joint in such a way as to obtain a similar number of samples in positive and negative classes. Good-quality joints were considered as positive class. Then, the sets and types of joints were combined to obtain one database used to build the neural network.

A summary of the developed networks, which differ in hyperparameters, such as the number of neurons in the hidden layer, activation function in the hidden layer, activation function in the output layer, learning algorithm, and error function, is presented in Table 5. The appropriate network for predicting the parameters of the FSW process was selected based on the following criteria:

1. The largest possible area under the curve (AUC), affecting the quality of the network;
2. The largest possible accuracy of the testing set, affecting the quality of the network;
3. The smallest possible number of neurons, affecting the level of computation complexity; and
4. Types of activation functions as simple as possible, affecting the level of complexity of calculations.

Based on the results presented in Table 5, it can be concluded that multi-layer perceptron (MLP) networks provide much better network quality (AUC of about 0.9) than radial basis function (RBF) networks (AUC of about 0.5).

The parameters of the MLP network were analyzed, and the learning algorithm was compared assuming that the remaining parameters were identical. The following optimization algorithms were considered: gradient descent (GD), conjugate gradient method (CG), and Broyden–Fletcher–Goldfarb–Shanno (BFGS). The learning rate for the conjugate gradient method was set to 0.1. The area under the curves (AUCs) for GD, CG, and BFGS were 0.858, 0.934, and 0.973, respectively. Although the AUC for the CG and BFGS algorithms was over 0.9, differences in accuracy were observed. The accuracies of the testing sets for GD, CG, and BFGS algorithms were 75.0%, 87.5%, and 93.5%, respectively. The network with the BFGS learning algorithm achieved the highest AUC and the highest accuracy, and it was further optimized.

The error function was then verified. Sum square error (SSE) and cross entropy were considered. Regardless of the adopted activation functions, networks with an error function estimated on the basis of cross entropy achieved an AUC of less than 0.8. Therefore, network optimization with SSE error function (AUC greater than 0.9) was selected for further analysis.

The influence of the number of neurons on the quality of the neural network for the classification was assessed. Networks with 16, 19, and 21 neurons in the hidden layer were considered. The network with 16 neurons in the hidden layer achieved the worst quality, with an AUC of 0.966. Networks with 19 and 21 neurons achieved similar quality, with AUCs of 0.973 and 0.972, respectively. The accuracies of the testing set in networks with 19 and 21 neurons were also similar at 93.5% and 92.3%, respectively. Based on the adopted evaluation criteria, it was decided to choose a less complex network with 19 neurons in the hidden layer.

Additionally, for the network with 19 neurons in the hidden layer, the impact of the adopted activation functions on the quality of the network was analyzed. The following activation functions were considered in the hidden and output layers: logistic and linear, logistic and hyperbolic tangent, and exponential and hyperbolic tangent. Due to the similar AUCs and accuracies of the testing sets of all three networks, the network with the simplest activation functions was selected.

**Figure 5:**
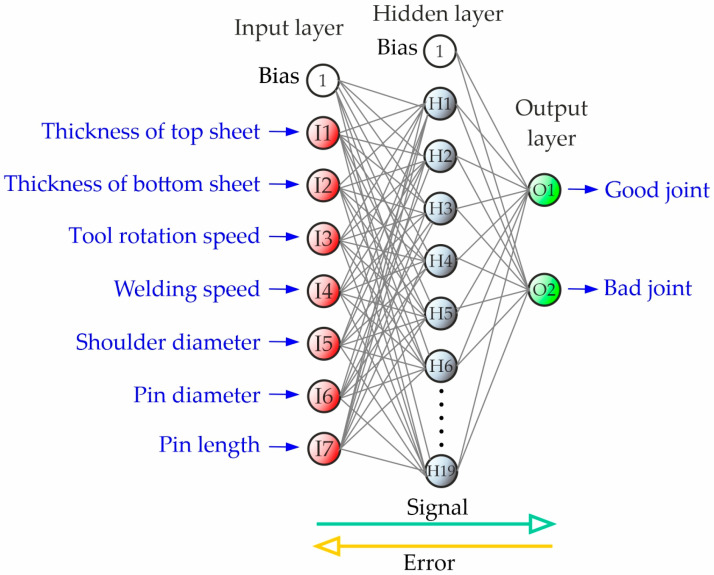
Structure of the designed artificial neural network for classification.

A neural network with a 7-19-2 topology was chosen for predicting welding parameters. The network consists of one input layer with 7 neurons, one hidden layer with 19 neurons, and one output layer with 2 neurons. The structure of the designed artificial neural networks for classification was presented in Figure 5. Neurons in the input layer corresponded to the following data from the experiment: thickness of the first sheet, thickness of the second sheet, welding speed, rotational speed, shoulder diameter, pin diameter, and pin length. Neurons in the output layer corresponded to the positive and negative classes. Classification was performed using multi-layer perceptron (MLP). Adjusting the weights and bias in the input and hidden layers were used as key factors to bring the results closer to the target and reduce the error rate. A logistic activation function in the hidden layer and a linear activation function in the output layer were used.

The Broyden–Fletcher–Goldfarb–Shanno (BFGS) optimization algorithm was used for teaching. It was assumed that the maximum training time would not exceed 300 epochs. This period was shortened when symptoms of network overlearning were detected. The network learning process was analyzed by observing the change in the value of errors in different learning epochs. The process was stopped before overfitting occurred. As a result, the error calculated for the test set, instead of no longer decreasing as the error of the training set decreased, began to increase. Sum square error was applied:

(1) $$SSE=\sum_{i=1}^{N}{\left(d_{i}-y_{i}\right)}^{2}$$

where

*d_i_*—dependent variable,
*y_i_*—predicted output.

The performance of classifier was mainly evaluated using the following evaluation parameters: area under the curve (AUC), receiver operating characteristics (ROC) curve, sensitivity, specificity, precision, negative predictive value, and accuracy. The mathematical representation of the evaluation index is as shown in the following equations:

(2) $$Sensitivity=\frac{TP}{TP+FN}$$

(3) $$Specificity=\frac{TN}{TP+FP}$$

(4) $$Precision=\frac{TP}{TP+FP}$$

(5) $$Negative\;predictive\;value=\frac{TN}{TN+FN}$$

(6) $$Accuracy=\frac{TP+TN}{TP+TN+FP+FN}$$

where

*Sensitivity* (true positive rate)—the probability of a positive test result, conditioned on the individual truly being positive.
*Specificity* (true negative rate)—the probability of a negative test result, conditioned on the individual truly being negative.
*Precision* (positive predictive value)—the fraction of relevant instances among the retrieved instances.
*Negative predictive values*—the proportion of negative results that are true negative results.
*Accuracy*—determines how close a given set of measurements are to their true value.
*TP*—true positive, a test result that correctly indicates the presence of a condition or characteristic.
*TN*—true negative, a test result that correctly indicates the absence of a condition or characteristic.
*FP*—false positive, a test result that wrongly indicates that a particular condition or attribute is present.
*FN*—false negative, a test result that wrongly indicates that a particular condition or attribute is absent.

**Figure 6:**
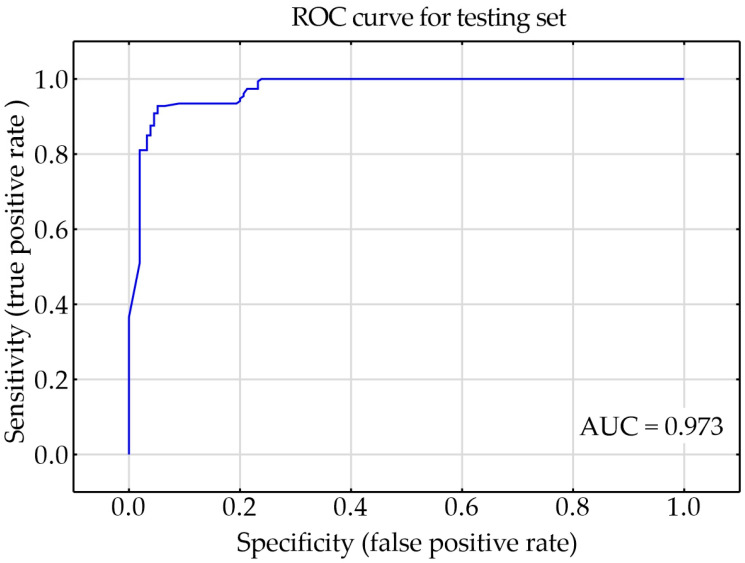
Receiver operating characteristics (ROC) curve for the testing set.

Only 72 epochs were needed to train the network. The receiver operating characteristics (ROC) curve for the testing set was shown in Figure 6. The AUC of the designed network was of 0.973. The quality of the testing set was approximately 93.5%.

The degree of efficiency of the neural network can be best assessed by analyzing its confusion matrix. Table 6 shows how many times the diagnoses provided by the network (“prediction class”) for the object specified as belonging to class 1 corresponded to the good-quality FSW joint (actual class = 1) and how many times the diagnosis prediction class = 0 corresponded with actual class = 0. The cases when prediction class = 0 corresponded with actual class = 1 and vice versa prediction class = 1 corresponded with actual class = 0 represented errors in the identification of a good-quality FSW joint. As shown in the confusion matrix for the testing set, the errors never exceeded 6%. This result was assessed as very satisfactory.

**Figure 7:**
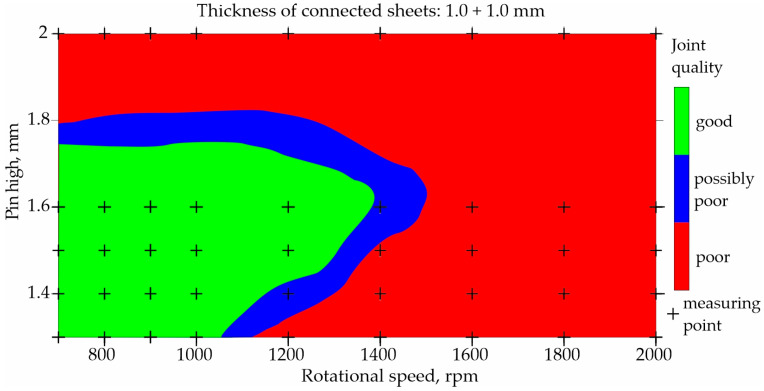
Map based on training data showing the influence of rotational speed and pin length on the quality of FSW joints made of two 1.0 mm thick sheets.

**Figure 8:**
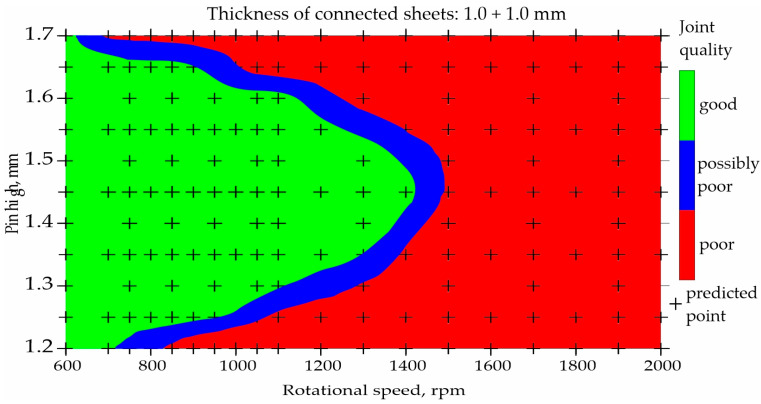
Map based on predictions showing the influence of rotational speed and pin length on the quality of FSW joints made of two 1.0 mm thick sheets.

**Figure 9:**
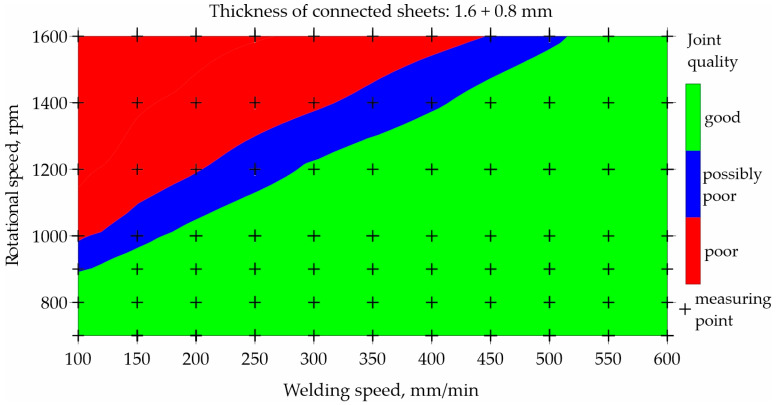
Map based on training data showing the impact of welding and rotational speed on the quality of FSW joints made of 1.6 and 0.8 mm thick sheets.

**Figure 10:**
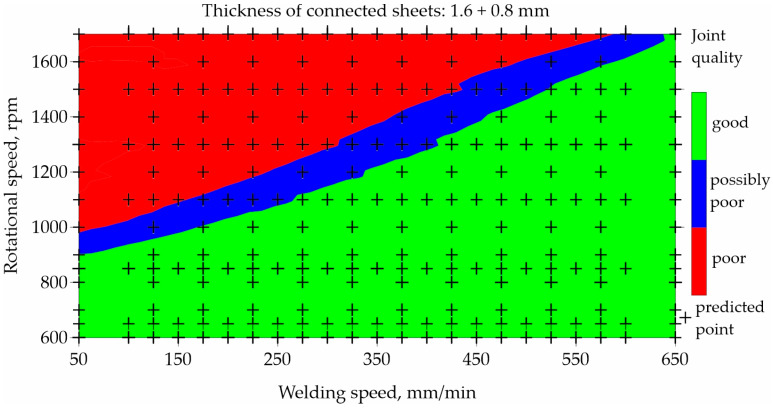
Map based on prediction showing the impact of welding speed and rotational speed on the quality of FSW joints made of 1.6 and 0.8 mm thick sheets.

To illustrate the prediction capabilities of the classification network, maps were built for representative types of joints and representative parameters of the FSW process, as shown in Figure 7, Figure 8, Figure 9 and Figure 10. An orthogonal grid generation model was developed by solving the governing equations of coordinate transformation with a local polynomial collocation method. The fitting method was applied only to selected types of joints and selected process parameters for which close to linear relationships could be observed. Nevertheless, it should be emphasized that the ANN method has a better accuracy in predicting datapoints than fitting method [13]. The use of the fitting method to present data obtained using ANNs introduced inaccuracies in relation to the values obtained using ANNs.

The classification of joints made of two 1.0 mm thick sheets is shown in Figure 7 and Figure 8. Both figures show the influence of rotational speed and pin height on the quality of the joints. The remaining parameters were constant: welding speed of 150 mm/min and shoulder and pin diameter of 7 mm and 2 mm, respectively. Figure 7 shows the map built on the basis of data from the experiment, while Figure 8 shows the map built using the developed neural network. The parameters for which poor-quality joints were obtained during welding are marked in red. Parameters that resulted in good-quality joints are marked in green. Blue indicates a possible poor joint quality. The places where black crosses appear indicate the welding parameters on the basis of which the maps were developed. The map in Figure 8 was built solely on the basis of predictions.

Analyzing the maps in Figure 7 and Figure 8, it can be observed that using the tool with a pin length of 1.25–1.65, it is possible to obtain good-quality joints at a relatively low rotational speed of up to approximately 900 rpm. When using a higher rotational speed, the tool with a longer pin of 1.35–1.6 mm is needed.

The critical rotational speed necessary to obtain good-quality joints is approximately 1400 rpm. The critical length of the pin is 1.7 mm. This is due to the amount of heat generated when joining two sheets of 1.0 mm thickness each.

Figure 9 and Figure 10 illustrate the influence of welding and rotational speed on the quality of FSW joints made of 1.6 and 0.8 mm thick sheets. Figure 9 shows the map created based on the experimental data used to train the neural network. Figure 10 shows the map resulting from the prediction of the neural network for classification. As in the previous example, poor-quality joints are marked in red, good-quality joints are marked in green, and blue indicates a possible poor quality. The results are presented for the samples made using the T12 tool. The welding speed in the network learning set was in the range of 100–600 mm/min, and the rotational speed was in the range of 700–1600 rpm.

The prediction was developed for an extended range of welding speed from 50 to 650 mm/min and a rotational speed in the range of 600–1700 rpm. The prediction was per-formed only for data on which the network was not trained and is marked with black crosses. Analyzing the welding parameters generating good-quality joints made of 1.6 and 0.8 mm thick sheets, it can be seen that it is possible to use any welding speed ranging from 50 to 650 mm/min, while using a low rotational speed up to 900 rpm.

Arbegast [26] proposed a fundamental relationship (Equation (1)) to establish a correlation between peak temperatures in welding and the primary parameters of the FSW process. This relationship also described the heat input index in work [27], in which the kissing bond phenomenon in AA5083-H112 friction stir butt welds was investigated in joints welded using a matrix of welding parameters, with tool rotation speeds of 800, 1000, and 1200 rpm and feed speeds 100, 200, and 300 mm/min. When considering a specific tool geometry and plunge depth, the maximum temperature primarily depends on the rotation rate, while the heating rate is influenced by the traverse speed. This relationship does not represent an analytical process model predicting trends based on the mechanics of the process. Instead, it serves as a tool to express the general impact of spindle speed and travel speed on peak temperatures during welding. The squared term involving spindle speed highlights the dominant effect of spindle speed on workpiece heating compared to the travel speed. As FSW is achieved through the combined effect of the tool feed rate and rotation speed, it is essential to consider their combined effect as well. In FSW, a semi-quantitative function, the heat input index (HI), can be expressed:

(7) $$HI=\frac{\omega^{2}}{\upsilon \cdot 10^{3}}$$

where

*ω*—spindle speed or tool rotation rate in rpm,
*ν*—feed rate or tool travel speed in mm/min.

It is widely recognized that the heat input index during FSW increases with the increase in the rotation rate and the decrease in the feed rate. Consequently, this leads to more favorable heat supply conditions, enhancing the efficacy of metal joining and the dispersion of potential oxide clusters during the FSW process.

**Figure 11:**
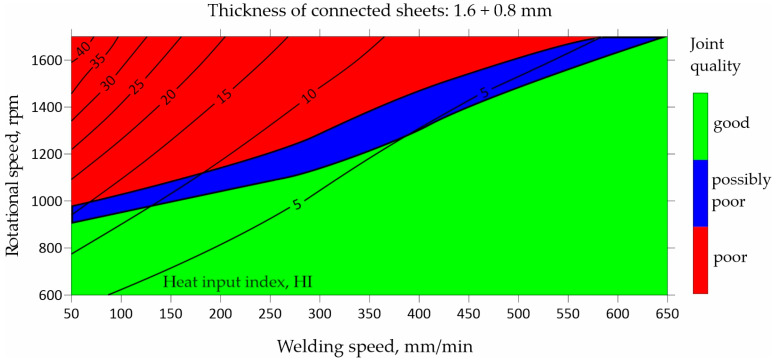
Map showing impact of welding speed and rotational speed on heat input index of FSW joints made of 1.6 and 0.8 mm thick sheets.

Figure 11 illustrates a relationship, which shows that by increasing the rotational speed above 900 rpm, the welding speed should also be increased. This is caused by the amount of heat generated by the tool. To obtain a good-quality FSW joint, it is essential to maintain a consistent heat supply to the sheets being welded. The amount of heat depends on the thickness of the joined sheets and the type of tool used. The specified level of heat can be achieved through a combination of a slow welding speed and a relatively low rotational speed. Alternatively, the same heat level can be attained by increasing both the welding speed and the rotation speed.

### 3.2. Regression

To predict the load-bearing capacity of the joint, another neural network was built for regression. To develop the network, similar to the classification network, the same samples were used, i.e., a total of 608 samples. The same sample labelling as in the classification network was used to assign samples to the learning, testing, and validation sets as shown in Table 4.

A summary of the developed networks for classification is presented in Table 7. The analyzed ANNs differ in hyper-parameters, such as the number of neurons in the hidden layer, activation function in the hidden layer, activation function in the output layer, learning algorithm, and error function. The selection of the appropriate network for predicting the load-bearing capacity of the FSW samples was assessed based on the following criteria:

1. The largest possible accuracy of the testing set, affecting the quality of the network;
2. The smallest possible error of the testing set, affecting the network quality;
3. The smallest possible number of neurons, affecting the level of computation complexity; and
4. Types of activation functions as simple as possible, affecting the level of complexity of calculations.

The first comparison was between multi-layer perceptron and radial basis function networks. The accuracy of the testing set of the RBF was about 31%, but a greater value of 90% was obtained for the MLP. Thus, the multi-layer perceptron network was chosen for further consideration.

Then, the parameters of the MLP network were analyzed. The learning algorithm was compared. The gradient descent (GD), conjugate gradient method (CG), and Broyden–Fletcher–Goldfarb–Shanno (BFGS) optimization algorithms were considered. The learning rate for conjugate gradient method was equal to 0.1. The accuracies of the testing sets for GD, CG, and BFGS were 87.9%, 39.2%, and 92.5%, respectively. The network with the BFGS learning algorithm achieved the highest accuracy, and it was further optimized.

The impact of the number of neurons on the performance of the neural network for the regression was also assessed. The networks with 16, 19, and 21 neurons in the hidden layer were considered. The networks with 16 and 21 neurons in the hidden layer demonstrated similar performance level, with testing set accuracies of 90.3% and 90.1%, respectively. The errors of testing sets for both networks were equal to 0.44 and 0.45. The highest accuracy was achieved by the network with 19 neurons in hidden layer. Its testing set accuracy was 92.5%, and the error of the testing set was of 0.35. Based on these results, the network with 19 neurons was selected as the most effective.

Furthermore, the impact of the activation functions on the quality of the network with 19 neurons in the hidden layer was analyzed. The following activation functions were considered in the hidden and output layers: hyperbolic tangent and linear, logistic and hyperbolic tangent, and logistic and linear. The network with hyperbolic tangent and linear functions achieved the highest accuracy of the testing set of 92.5%. The accuracies of the remaining two pairs of functions were 89.5% and 90.9%, respectively. The network with fewer complex functions, i.e., logistic and linear, achieved lower accuracy than the network with hyperbolic tangent and linear functions. Therefore, the network with hyperbolic tangent function in the hidden layer and linear function in the output layer was chosen for the regression.

**Figure 12:**
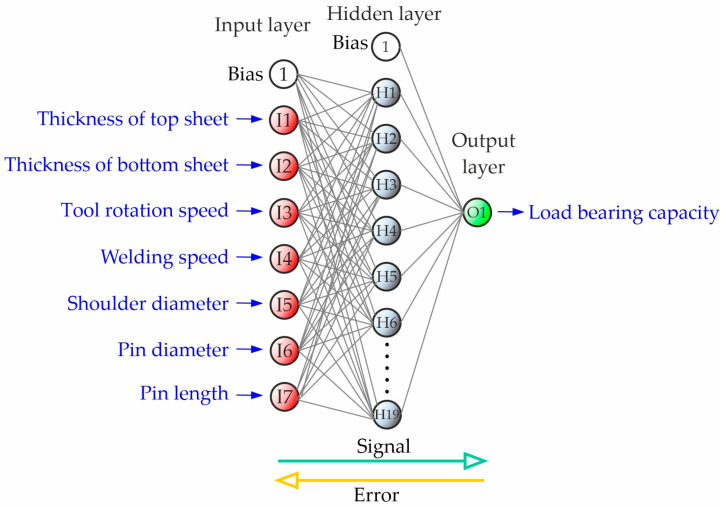
Structure of the designed artificial neural network for regression.

A neural network with a 7-19-1 topology was used for prediction of load-bearing capacity of the FSW samples. This means that the network had an input layer with 7 neurons, one hidden layer with 19 neurons, and an output layer with 1 neuron (Figure 12). Neurons in the input layer corresponded to the following data from the experiment: thickness of the first sheet, thickness of the second sheet, welding speed, rotational speed, diameter of shoulder, and diameter and length of the pin. The neuron in the output layer corresponded to the load-bearing capacity of the FSW joint.

**Figure 13:**
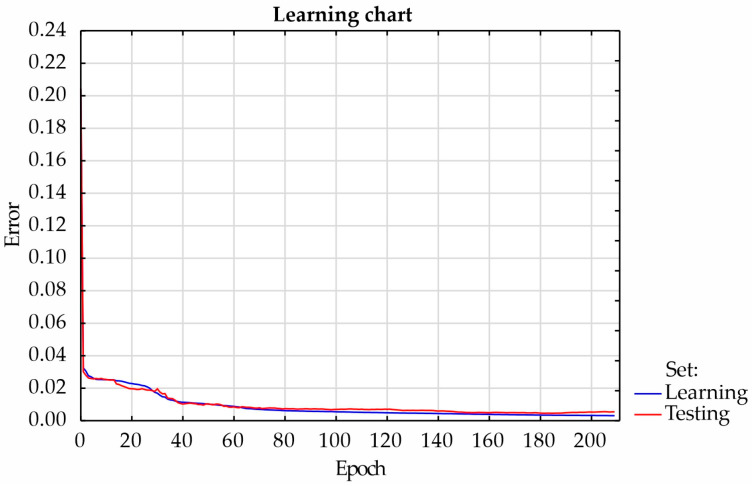
The process of learning of the regression artificial neural network.

**Figure 14:**
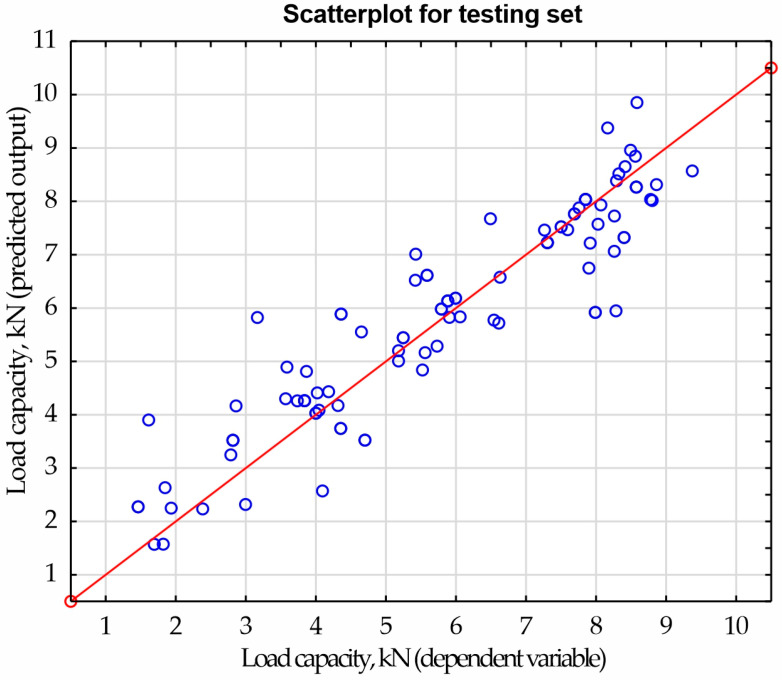
Scatter plot for testing set.

Regression was performed using multi-layer perceptron. Adjusting the weights and bias in the input and hidden layers was used to reduce the error rate. A hyperbolic tangent activation function was used in the hidden layer, and a linear activation function was used in the output layer. Learning was performed using the BFGS optimization algorithm. It was assumed that the maximum training time would not exceed 200 epochs. As in the case of classification, the regression network learning process was assessed by observing the change in SSE error values. Analyzing the graph of the artificial neural network learning process (Figure 13), it was found that 184 epochs were necessary to train the network. Thanks to this, the network had sufficient approximation and generalization ability. The accuracy of the testing set was approximately 92.5%. However, Figure 14 shows a scatter plot between the dependent variable and the predicted response of load-bearing capacity, which helped determine the quality of the model.

**Figure 15:**
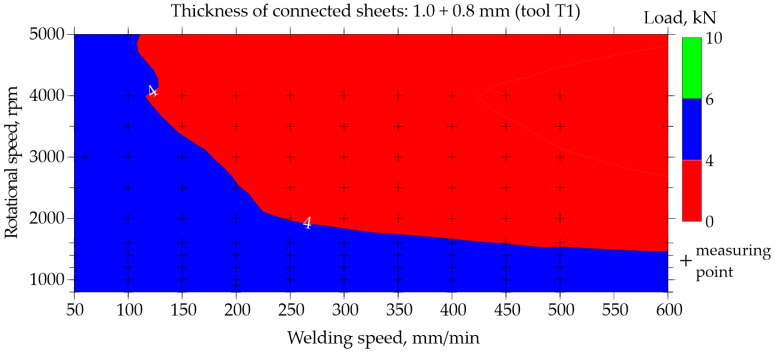
Map based on training data showing the impact of welding speed and rotational speed on the load-bearing capacity of FSW joints made of 1.0 and 0.8 mm thick sheets (tool T1).

**Figure 16:**
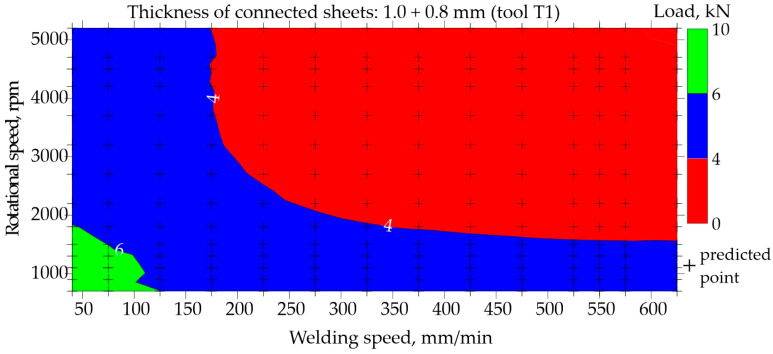
Map based on prediction showing the impact of welding speed and rotational speed on the load-bearing capacity of FSW joints made of 1.0 and 0.8 mm thick sheets (tool T1).

**Figure 17:**
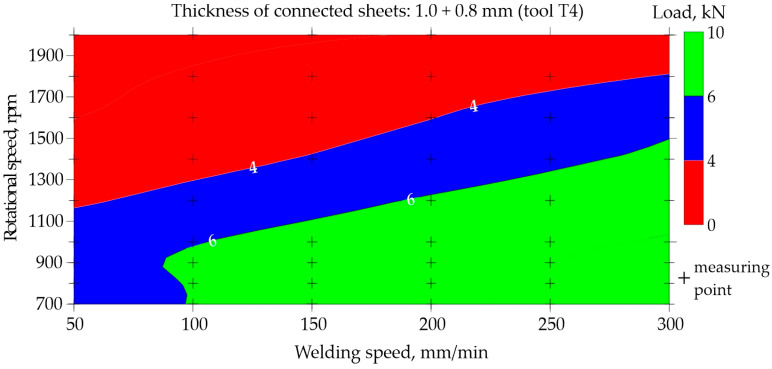
Map based on training data showing the impact of welding speed and rotational speed on the load-bearing capacity of FSW joints made of 1.0 and 0.8 mm thick sheets (tool T4).

**Figure 18:**
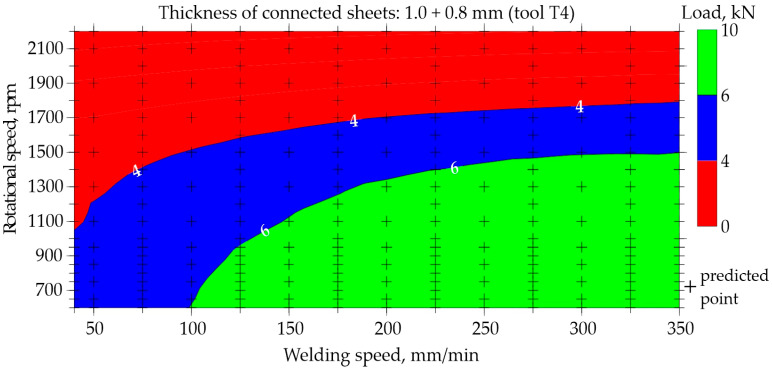
Map based on prediction showing the impact of welding speed and rotational speed on the load-bearing capacity of FSW joints made of 1.0 and 0.8 mm thick sheets (tool T4).

The possibility of predicting the neural network for regression was presented based on data for the representative type of joint made of 1.0 and 0.8 mm thick sheets. Maps illustrating the relationship between the welding speed and rotational speed for tools T1 and T4 are shown in Figure 15, Figure 16, Figure 17 and Figure 18. The grid generation model was developed using the local polynomial method.

Maps for assessing the load-bearing capacity of joints made using the T1 tool with a shoulder diameter of 7 mm and a pin diameter and length of 2 mm and 1.2 mm, respectively, are shown in Figure 15 and Figure 16. Figure 15 shows the map based on experimental data that were used to train the network. Load capacity equal to or greater than 6.0 kN is marked in green. It can be interpreted as the load capacity required by the joints since the minimum acceptable load capacity is 5.8 kN. As can be seen from Figure 15, no areas marked in green were observed. However, it can be observed that when welding joints using the T1 tool, the highest load-bearing capacity is achieved by joints welded at a low rotational speed up to 1600 rpm.

The map resulting from the network prediction is shown in Figure 16. The results are presented for an enlarged area of welding speed in the range of 40–650 mm/min and rotational speed in the range of 700–5200 rpm. Using the ability to approximate and generalize the constructed neural network, a small area marked in green was observed in Figure 16, which means it is possible to weld FSW joints using the T1 tool in such a way that their load capacity exceeds 6.0 kN. For this purpose, a low welding speed of 40–50 mm/min and a low rotational speed of approximately 1600 rpm should be used.

Both Figure 15 and Figure 16 show that FSW welding using the T1 tool with a high welding speed and high rotational speed results in joints with a very low load-bearing capacity not exceeding 4.0 kN.

Maps for assessing the load-bearing capacity of joints made using the T4 tool with a shoulder diameter of 10 mm and a pin diameter and length of 4 mm and 1.86 mm, respectively, are shown in Figure 17 and Figure 18. Experimental data were used to prepare Figure 17. It is easy to observe a large area of FSW process parameters, which are used to create joints with a load capacity exceeding 6.0 kN. The best joints transfer a force of approximately 8.5 kN. To make such joints, it is necessary to set a low rotational speed of 700–1000 rpm and a fairly high welding speed of at least 200 mm/min. Reducing the welding speed below 150 mm/min and simultaneously increasing the rotational speed above 1300 rpm results in a significant reduction in the load-bearing capacity of the joints to below 4.0 kN, which is marked in red.

Very similar relationships were observed in Figure 18, which was developed on the basis of neural network predictions. Even taking into account the extended range of welding speeds of 40–350 mm/min and rotational speeds of 500–2200 rpm, it was found that the highest load-bearing capacity of the joints is achieved using a relatively high welding speed and low rotational speed. The developed maps (Figure 15, Figure 16, Figure 17 and Figure 18) also allow for the assessment of the impact of the tool itself on the quality of joints. FSW joints made using the T4 tool with a larger shoulder diameter of 10 mm show a much higher load capacity of approximately 8.5 kN than joints made using the T1 tool, in which the shoulder diameter is smaller at 7 mm. The load capacity of joints welded using the T1 tool is approximately 6.0 kN at most.

When using the T1 tool with shoulder and pin diameters of 7 and 2 mm, respectively, to obtain the joint with an acceptable load-bearing capacity, a low welding speed of 40–50 mm/min and a low rotational speed of approximately 700–1600 rpm should be used. However, using a larger tool with shoulder and pin diameters of 10 and 4 mm, respectively, the best joints are obtained by conducting the FSW welding process at a higher welding speed ranging from 200 to 300 mm/min, coupled with a similarly low rotational speed of 700 to 1000 rpm. The usage of a larger tool with an expanded shoulder diameter results in increased heat accumulation within the welded material, necessitating the use of a higher welding speed. It should also be taken into account that the T1 tool had a pin length of 1.2 mm, and the T4 tool had a pin length of 1.86 mm. The longer pin leads to the larger volume of mixed materials, thereby improving the quality of the FSW joint.

## 4. Case Study

This work uses a case study as a research methodology to examine a specific joint in the broader context of neural network applications and thus draw more general conclusions. The knowledge obtained from the case study will be used to better understand the FSW process and to improve real actions. The basic parameters of the FSW process, thanks to which the joint was made, were a rotational speed n = 700 rpm and a welding speed v = 100 mm/min. The choice of the J1 joint as a case study resulted from the relatively large problems occurring when joining thin sheets using the FSW method. The parameters for making the joint were selected based on the charts in Figure 17 and Figure 18. The map prepared on the basis of training data in the area of parameters n = 700 rpm and v = 100 mm/min showed a slight discrepancy with the map based on prediction.

**Figure 19:**
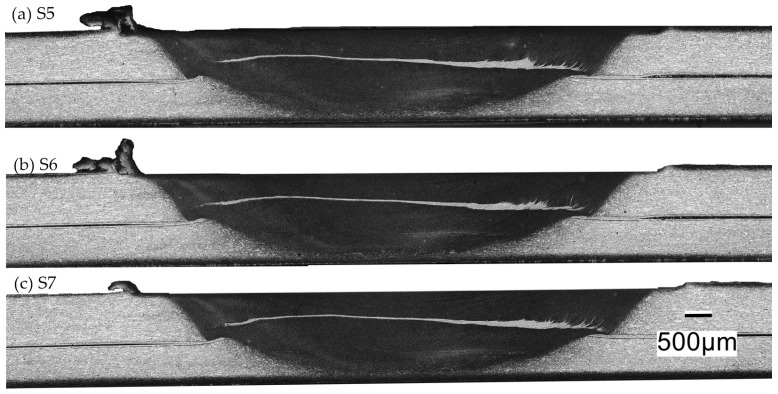
Macrostructure of (**a**) S5; (**b**) S6; and (**c**) S7 samples.

Figure 19 presents the macrostructure of the J1 joint across three different sections. The places where specimens S5, S6, and S7 were taken from the FSW joint are shown in Figure 2. The macrostructures do not show noticeable structural defects. The lighter area corresponds to the base material, while the darker area represents the stir zone. A line of aluminum clad is visible in the center of the stir zone. In [28], it was indicated that ultrasonic testing enables the classification of joints into the following categories: joints without material discontinuities, joints with material discontinuities on the advancing side, and joints with discontinuities extending across the entire width of the stirring zone. The tests focused mainly on horizontally oriented defects.

Modelling of the FSW process allows insight into the complex phenomena occurring in this process. Finite element analysis models can predict the likelihood of defects, such as voids, inclusions, or improper material stirring in a welded joint. In the current version of the neural network, the heat input index has not been included, but further work will lead to its inclusion, which will provide the neural network with new forecasting capabilities.

**Figure 20:**
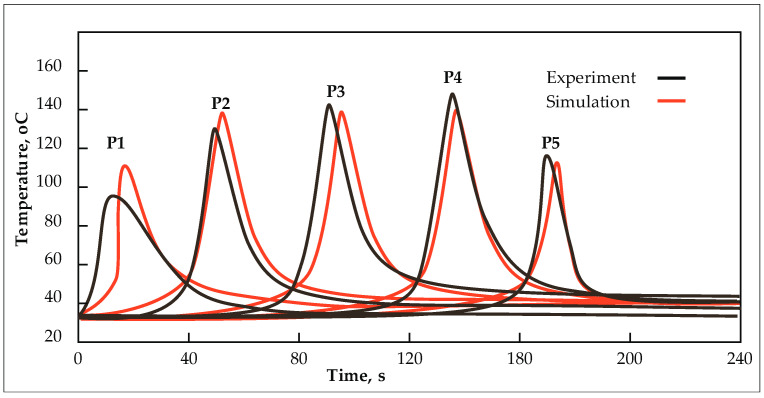
Comparison of experimentally measured (black) and numerically calculated (red) temperature values at measurement points for joint type J1. Basic process parameters: rotational speed of n = 700 rpm, welding speed v = 100 mm/min, and tool T4.

Figure 20 presents a comparative analysis of temperature measurements obtained from experimental data and those calculated using the finite element method. The numerical model employed in this study is explained in the authors’ prior works, notably [29]. In this work, the thermal model considered heat conduction and convection allocated to pertinent structural elements. Temporal functions were employed to characterize the movement of the heat source. The numerical model incorporated both the welding and clamp release stages. The simulation output encompasses the temperature field, residual stresses, and deformations manifesting within the welded structure. The maximum discrepancy between the calculated and measured temperature peaks is within 14%, specifically for the thermocouple positioned at point P1. Discrepancies between experimental measurements and numerical simulations arise from challenges associated with accurately determining the thermocouple positions during the experiment and variations in process parameters attributable to frictional resistance encountered in the process.

**Figure 21:**
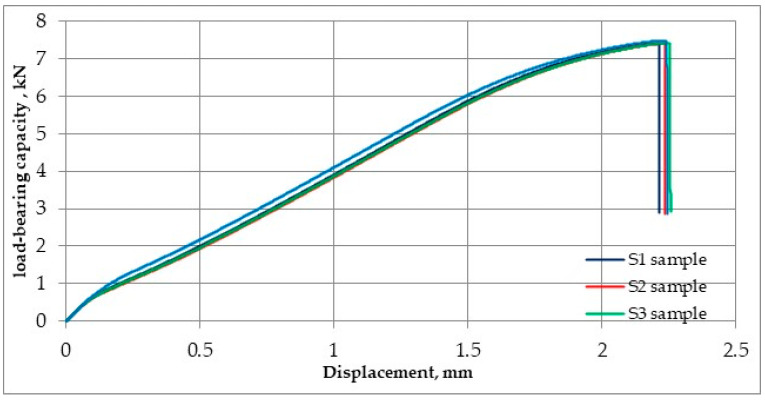
Load capacity vs. displacement graph for S1–S4 samples.

The predictive capabilities of the developed neural network model based on the map shown in Figure 18 indicate that the load-bearing capacity of the joint J1 is in the range of 4–6 kN. The exact result predicted by the designed ANN was 6.22 kN. However, the static tensile test performed for four samples S1–S4, cut from joint J1 according to the diagram in Figure 2, showed that the averaged load capacity of the joint is 7.42 kN, as shown in Figure 21. The tests carried out showed that the neural network model predicted a lower load-bearing capacity of the joint than the tests showed. Neural networks, despite their effectiveness, are subject to inherent limitations and potential inaccuracies. Common challenges and sources of inaccuracy in neural networks often emanate from the quality and quantity of the available data. The quality of data, including its relevance, accuracy, and representativeness, significantly influences the model’s ability to discern pertinent features and generalize effectively. In instances where data inadequacies prevail, models may encounter difficulties in capturing the underlying patterns, leading to compromised performance. Addressing these challenges necessitates a comprehensive understanding of the data intricacies and the implementation of strategies to enhance the robustness and generalization capabilities of neural network models in diverse contexts. It is worth noting that an orthogonal grid generation model was developed by solving the governing equations of coordinate transformation with a local polynomial collocation method. This introduced further inaccuracies into the results obtained using the artificial neural network. From the point of view of joint design, it is advantageous if the predicted load capacity of the joint is lower than the real value. It would be unacceptable if the predicted load capacity was less than the value obtained from experience.

Additionally, it should be taken into account that the FSW process is sensitive to the accuracy of execution, which largely depends on the employee’s skills. The quality of the joints is influenced by several factors, including the shift in which production was carried out (the joints were made in three shifts) and the method of fastening and pressing the joined sheets. These factors are difficult to measure and were not declared as a neuron with input data. The neural network was trained on the large database in which the previously mentioned factors influenced the quality of joints.

**Figure 22:**
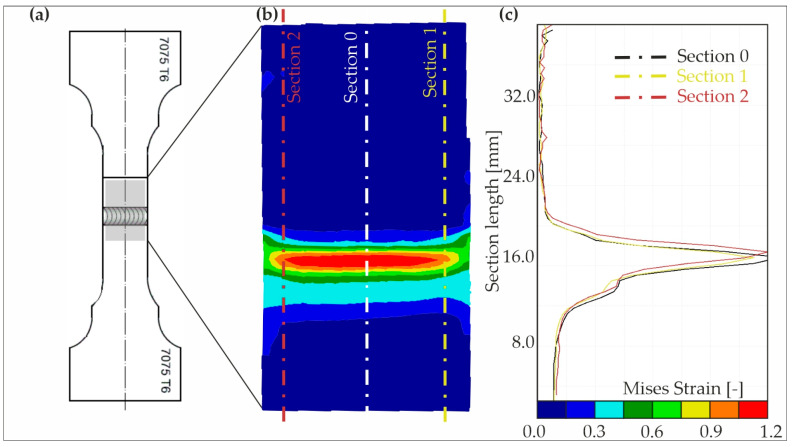
Mises strain on a sample before rupture; (**a**) sample S4; (**b**) strain in the measuring field and (**c**) strain on cross-sections 0–2.

Tensile tests were recorded using a GOM Aramis system for non-contact real-time analysis of specimen deformation. The system consists of two cameras and two lamps that illuminate the measurement area and a computer workstation with special software that allows digital correlation of the recorded images. The Aramis system analyses the displacements of the measuring points from which it calculates the values of Mises strain field occurring in the sample due to the load applied. Within this context, there is intention to employ the Mises strain field of the fracture zone for training neural networks in forthcoming research. Figure 22 shows the Mises strain field on a sample before rupture, obtained when stretching sample S4 in a tensile test. The utilization of this strain field as a training dataset for neural networks is anticipated to provide valuable insights into the relationship between material deformation and fracture behavior.

The application of such a dataset in neural network training holds promise for enhancing the predictive capabilities of models focused on fracture prediction and related mechanical properties. This approach aligns with the broader objective of leveraging advanced computational techniques to deepen our understanding of material behavior under stress conditions, ultimately contributing to advancements in fracture mechanics and structural integrity assessments.

## 5. Discussion

The FSW process is complex and characterized by non-linear relationships between process parameters and the properties of welded joints. Machine learning models, especially those based on deep neural networks, are capable of modelling non-linear and irregular dependencies, allowing for precise mapping of the FSW process. Additionally, FSW processes are often subject to multiple criteria, such as strength, corrosion resistance, and fatigue strength. Machine learning algorithms, especially those dedicated to multi-criteria optimization, can effectively take into account and optimize various criteria simultaneously. Industrial processes, including FSW, may be subject to changing conditions, such as tool wear or changes in raw materials. Machine learning models are flexible and adaptable to new conditions, allowing optimization to remain effective in changing conditions.

Traditional optimization methods can require a lot of trial and error, which is time-consuming and expensive. Machine learning models, once trained, can predict results for different sets of process parameters, speeding up the optimization process. FSW processes are often complex and difficult to understand at an analytical level. Machine learning models help identify complex relationships between process parameters and joint properties, leading to more effective optimizations. Machine learning algorithms can be integrated into the production process to automate the optimization of the process, reducing the need for human intervention and increasing efficiency.

To optimize the FSW process using machine learning, it is important to properly prepare training data and adapt the models to the specific nature of the process and production expectations. However, properly configured machine learning models can effectively accelerate and improve the optimization of FSW processes.

Experimental data are very valuable, but their acquisition is often time-consuming and expensive. Machine learning, including the use of artificial neural networks, can be an effective tool for searching for optimal process parameters, including data regarding the FSW process. Machine learning allows the prediction of results for various combinations of parameters without testing them directly. The presented ANN model is able to predict the results of the FSW process quite accurately based on training data, which makes it an economical alternative that allows for shortening the time of obtaining experimental data and reducing the associated costs.

The problem of optimizing FSW parameters is very complex, with many variables and non-linear dependencies. The authors’ experience shows that ML, and especially ANNs, cope with this problem better than traditional methods. Ultimately, the ML model is to be used in industrial conditions, so it must be simple enough to use in practice and adapted to the specific nature of the FSW process.

The use of ANNs in the analysis of the FSW process may have several sensible applications related to the characteristics and complexity of this welding process. Below are some potential benefits:

- Nonlinearity Modelling: The FSW process is nonlinear and complex, with many variables influencing its results. Artificial neural networks can effectively model complex, non-linear relationships between process parameters and welding results.
- Adaptation to Experimental Data: Due to the ability of ANNs to learn from data, they can be adapted to specific experimental data from the FSW process, enabling precise modelling of process characteristics.
- Process Optimization: ANNs can be used to optimize FSW process parameters. By training the network on experimental data and using it to predict results for various combinations of parameters, optimal welding conditions can be identified.
- Defect Diagnostics and Prediction: ANNs can be used to diagnose the FSW process and predict potential defects. Based on input data (process parameters) and results, the network can indicate areas where quality problems may occur.
- Adaptive Process Control: Using ANNs to monitor the FSW process in real-time and adjust parameters on the fly can improve weld quality and process efficiency.

## 6. Conclusions

By integrating the knowledge gained from the case study into the training processes of neural networks, there exists a promising avenue to enhance the predictive capabilities and efficacy of these computational models. The nuanced understanding of the FSW process, garnered through the analysis, is expected to provide valuable inputs for developing and fine-tuning neural network architectures specifically fitted for applications related to FSW processes. Based on the experience obtained in this work, the following conclusions can be drawn:

1. The selected parameters of FSW process, including rotational speed, welding speed, and joint and tool geometry, which were used as input data for training a neural network, allowed a correctly predicting neural network to be obtained.
2. The classification network was trained in just 72 epochs. The AUC of the network was of 0.973. The quality of the validation set was approximately 93.6%. In the confusion matrix for testing set, the errors never exceeded 6%. MLP 7-19-2 topology was used to build the regression network. This result was assessed as satisfactory.
3. The regression network was trained in only 184 epochs. The quality of the validation set was approximately 87.1%. MLP 7-19-1 topology was used to build the regression network. This result was also assessed as satisfactory.
4. The developed prediction maps (Figure 8, Figure 10, Figure 16 and Figure 18) allow for the selection of optimal parameters of the FSW process for three types of aluminum joints (EN AW 7075) and the assessment of the impact of the tool on the quality of the joints.
5. The case study showed that for the analyzed joint J1, neural networks predicted a 19% lower joint load-bearing capacity than the research showed. This is a safe approach from the design point of view.

This work aims to increase the use of artificial intelligence in materials engineering, with particular emphasis on the optimization and automation of complex processes, such as FSW. Moreover, the synergistic relationship between the insights obtained and neural network training can catalyze advancements in the practical implementation of artificial intelligence in the optimization and control of FSW processes. This approach can not only improve the precision and efficiency of FSWs, but also contribute to the broader development of smart manufacturing systems.

## Figures and Tables

**Table 1:** Configuration of analyzed joint types.

Joint Type	Top Sheet Metal Thickness t_t_ [mm]	Bottom Sheet Metal Thickness t_b_ [mm]
J1	1.0	0.8
J2	1.0	1.0
J3	1.6	0.8

**Table 2:** Chemical composition of analyzed sheet.

Content (wt.%)									
Cu	Mg	Cr	Zn	Ti	Mn	Si	Fe	Other	Al
1.5	2.6	0.2	5.4	0.3	0.2	0.3	0.4	0.11	residue

**Table 3:** Dimensions of the working parts of FSW tools.

Tool Type	Shoulder Diameter, ds [mm]	Pin Diameter, dp [mm]	Pin Height, hp [mm]
T1	7.0	2.0	1.20
T2	5.0	2.0	1.20
T3	1.6	0.8	1.10
T4	10.0	4.0	1.20
T5	10.0	4.0	1.26
T6	10.0	4.0	1.56
T7	10.0	4.0	1.30
T8	10.0	4.0	1.40
T9	10.0	4.0	1.50
T10	10.0	4.0	1.60
T11	10.0	4.0	1.84
T12	10.0	4.0	1.86
T13	10.0	4.0	1.88
T14	10.0	4.0	1.90

**Table 4:** Database for classification and regression.

Type of Joint (Thicknesses of Sheets)	Number of Samples from Experiment	Learning Set	Testing Set	Validation Set
1.0 mm + 0.8 mm	282	198	42	42
1.0 mm + 1.0 mm	228	160	34	34
1.6 mm + 0.8 mm	98	68	15	15

**Table 5:** Summary of the analyzed artificial neural network for the classification.

Network Type	Number of Neurons in the Hidden Layer	Activation Function in the Hidden Layer	Activation Function in the Output Layer	Learning Algorithm	Error Function	AUC	Accuracy of Learning Set	Accuracy of Testing Set	Accuracy of Validation Set	Number of Epochs
RBF	19	Gaussian	Linear	RBFT	SSE	0.506	61.7%	63.5%	61.3%	-
MLP	19	Logistic	Linear	GD	SSE	0.858	71.4%	75.0%	64.5%	173
MLP	19	Logistic	Linear	CG	SSE	0.934	85.8%	87.5%	79.6%	127
MLP	19	Logistic	Softmax	BFGS	Cross Entropy	0.673	51.1%	50.0%	38.7%	-
MLP	19	Exponential	Softmax	BFGS	Cross Entropy	0.722	51.1%	50.0%	38.7%	-
MLP	16	Logistic	Linear	BFGS	SSE	0.966	90.1%	91.3%	91.4%	62
MLP	19	Logistic	Linear	BFGS	SSE	0.973	93.2%	93.5%	93.6%	72
MLP	21	Logistic	Linear	BFGS	SSE	0.972	92.1%	92.3%	88.2%	84
MLP	19	Logistic	Hyperbolic tangent	BFGS	SSE	0.974	91.8%	90.4%	87.1%	55
MLP	19	Exponential	Hyperbolic tangent	BFGS	SSE	0.972	89.9%	93.3%	90.3%	64

**Table 6:** Confusion matrix for the testing set.

Prediction	Actual			
		Positive	Negative	
	Positive	141	8	Precision: 94.6%
	Negative	12	147	Negative predictive value: 92.5%
		Sensitivity: 92.2%	Specificity: 94.8%	Accuracy: 93.5%

**Table 7:** Summary of analyzed artificial neural networks for the regression.

Network Type	Number of Neurons in the Hidden Layer	Activation Function in the Hidden Layer	Activation Function in the Output Layer	Learning Algorithm	Error Function	Accuracy of Learning Set	Accuracy of Testing Set	Error of Testing Set	Accuracy of Validation Set	Number of Epochs
RBF	19	Gaussian	Linear	RBFT	SSE	29.4%	31.4%	5.1 × 10^9^	39.2%	-
MLP	19	Hyperbolic tangent	Linear	GD	SSE	85.6%	87.9%	0.55	82.2%	284
MLP	19	Hyperbolic tangent	Linear	CG	SSE	36.6%	39.2%	2.71	31.0%	4
MLP	16	Hyperbolic tangent	Linear	BFGS	SSE	92.9%	90.3%	0.44	88.3%	179
MLP	19	Hyperbolic tangent	Linear	BFGS	SSE	94.8%	92.5%	0.35	87.1%	184
MLP	21	Hyperbolic tangent	Linear	BFGS	SSE	93.5%	90.1%	0.45	84.9%	160
MLP	19	Logistic	Hyperbolic tangent	BFGS	SSE	93.8%	89.5%	0.49	80.0%	244
MLP	19	Logistic	Linear	BFGS	SSE	92.0%	90.9%	0.41	87.1%	159

## Data Availability

The data presented in this study are available on request from the corresponding author.
